# Draft Genome Sequence of *Salmonella enterica* subsp. *enterica* Serotype Saintpaul Strain S-70, Isolated from an Aquatic Environment

**DOI:** 10.1128/genomeA.01016-13

**Published:** 2013-12-12

**Authors:** Mitzi Estrada-Acosta, Andrés Medrano-Félix, Maribel Jiménez, Bruno Gómez-Gil, Josefina León-Félix, Luis Amarillas, Cristóbal Chaidez

**Affiliations:** Laboratorio de Microbiología Ambiental y de Alimentos, Centro de Investigación en Alimentación y Desarrollo, A.C., Unidad Culiacán, Culiacán, Sinaloa, Mexicoa; Facultad de Ciencias Químico-Biológicas de la Universidad Autónoma de Sinaloa, Ciudad Universitaria, Culiacán, Sinaloa, Mexicob; Centro de Investigación en Alimentación y Desarrollo, A.C., Unidad Mazatlán, Mazatlán, Sinaloa, Mexicoc; Instituto de Investigación Lightbourn, Chihuahua, Mexicod

## Abstract

*Salmonella* is a pathogen of worldwide importance, causing disease in a vast range of hosts, including humans. We report the genome sequence of *Salmonella enterica* subsp. *enterica* serotype Saintpaul strain S-70, isolated from an aquatic environment.

## GENOME ANNOUNCEMENT

*Salmonella* is one of the most important bacterial enteric pathogens worldwide (1) and is responsible for the great majority of intestinal and systemic infections in humans and other warm-blooded animals (2). Although the mammalian intestine tract is considered its natural habitat (3), *Salmonella* is constantly being shed into aquatic environments through human and animal feces (4). This pathogen has been detected in a wide range of natural settings, such as water (rivers, lakes, coastal waters, and sewage effluents) (4, 5), soil, and sediments, where it can survive for long periods (6, 7). The wide diversity of *Salmonella* serotypes and its survival capacity outside a definitive host contribute to its constant presence in aquatic environments (4).

*Salmonella enterica* subsp. *enterica* serotype Saintpaul is one of the most frequent causative agents of salmonellosis within the United States (8). Several fresh produce-borne outbreaks have been attributed to genomic variants within this serotype (9). In this study, an environmental strain of *S.* Saintpaul from the Laboratory for Environmental and Food Microbiology private collection was sequenced in order to determine the possible mechanisms related to its pathogenicity and capacity for adaptation to external environments.

*Salmonella* Saintpaul strain S-70 was sequenced using a next-generation sequencing (NGS) semiconductor-based technology platform (Ion Torrent Personal Genome Machine; Life Technologies) with a 316 chip. A total of 1.145 million reads were obtained (mean length, 156 bp), for a total of 179 Mbp and a coverage of 34.15×. The reads were assembled with MIRA version 3.4.0 to obtain 84 contigs (N_50_, 148.77 kbp). The contigs were further reassembled with Geneious R6 version 6.0.3, and the original contigs were annotated by RAST (10) (http://rast.nmpdr.org/).

The draft genome sequence of *Salmonella* Saintpaul S-70 is 4,624,379 bp, with an overall G+C content of 52.1%, 109 RNAs, 83 tRNAs, 4,775 coding sequences (CDSs), and 577 subsystems.

Genome analysis indicated that at least 281 genes might be related to cell wall functions, including genes for capsular and Gram-negative cell wall components. Also, we found 190 genes related to stress responses (e.g., osmotic and desiccation stress and cold and heat stress responses) and 203 genes associated with membrane transport (e.g., type II, III, IV, VI, and VII secretion systems) that allow for the export of proteins from within the bacterial cell to the extracellular milieu, for the exchange of genetic material with other bacteria, and for the transportation of virulence factors across the cell envelope.

The genome sequence revealed diverse genes that encode virulence factors (e.g., pili, lipopolysaccharides, flagella, fimbriae, capsular polysaccharide biosynthesis and assembly, type II and IV secretion systems, and siderophores), including genes that belong in *Salmonella* pathogenicity islands (SPIs). The genome sequence also revealed genes for several hypothetical proteins and genes with roles in DNA and RNA metabolism.

### Nucleotide sequence accession numbers.

*Salmonella* Saintpaul S-70 was obtained from the collection of the Laboratory for Environmental and Food Microbiology at CIAD, Culiacán, Mexico. This whole-genome shotgun project has been deposited at DDBJ/EMBL/GenBank under the accession no. AQQN00000000. The version described in this paper is the first version, AQQN01000000.
